# Research Progress of Traditional Chinese Medicine in Treatment of Myocardial fibrosis

**DOI:** 10.3389/fphar.2022.853289

**Published:** 2022-06-08

**Authors:** Chunzhen Ren, Kai Liu, Xinke Zhao, Huan Guo, Yali Luo, Juan Chang, Xiang Gao, Xinfang Lv, Xiaodong Zhi, Xue Wu, Hugang Jiang, Qilin Chen, Yingdong Li

**Affiliations:** ^1^ School of Traditional Chinese and Western Medicine, Gansu University of Chinese Medicine, Lanzhou, China; ^2^ Affiliated Hospital of Gansu University of Chinese Medicine, Lanzhou, China; ^3^ Gansu Provincial People’s Hospital, Lanzhou, China; ^4^ The Second Hospital of Lanzhou University, Lanzhou, China

**Keywords:** traditional Chinese medicine, myocardial fibrosis, research progress, mechanism, prevention and treatment

## Abstract

Effective drugs for the treatment of myocardial fibrosis (MF) are lacking. Traditional Chinese medicine (TCM) has garnered increasing attention in recent years for the prevention and treatment of myocardial fibrosis. This Article describes the pathogenesis of myocardial fibrosis from the modern medicine, along with the research progress. Reports suggest that Chinese medicine may play a role in ameliorating myocardial fibrosis through different regulatory mechanisms such as reduction of inflammatory reaction and oxidative stress, inhibition of cardiac fibroblast activation, reduction in extracellular matrix, renin-angiotensin-aldosterone system regulation, transforming growth Factor-β1 (TGF-β1) expression downregulation, TGF-β1/Smad signalling pathway regulation, and microRNA expression regulation. Therefore, traditional Chinese medicine serves as a valuable source of candidate drugs for exploration of the mechanism of occurrence and development, along with clinical prevention and treatment of MF.

## Introduction

Myocardial fibrosis (MF) is characterized by pathological changes in the extracellular matrix of myocardial cells where cardiac fibroblasts are activated and proliferated excessively, resulting in excessive accumulation of collagen fibres, excessive increase in the collagen content, and a significant increase in collagen volume (Numaguchi et al., 2011). The main pathological changes involved in MF are the increase in myocardial stiffness, decrease in myocardial contraction and relaxation ability, and insufficiency of coronary blood supply. MF is a common pathological manifestation of many cardiovascular diseases after their development to a certain stage. The disease is characterized by rapid onset, high mortality, and unknown mechanism, and effective treatments for the disease are lacking in modern medicine (Leask, 2010; Francesca et al., 2021). Moreover, the treatment cost is high. In recent years, a large number of studies have been conducted on the prevention and treatment of MF by using traditional Chinese medicine. The treatment cost and side effects of traditional Chinese medicine are low. Therefore, it has garnered considerable attention of scholars at both home and abroad.

## Myocardial Fibrosis Mechanism According to Modern Medicine

**FIGURE 1:**
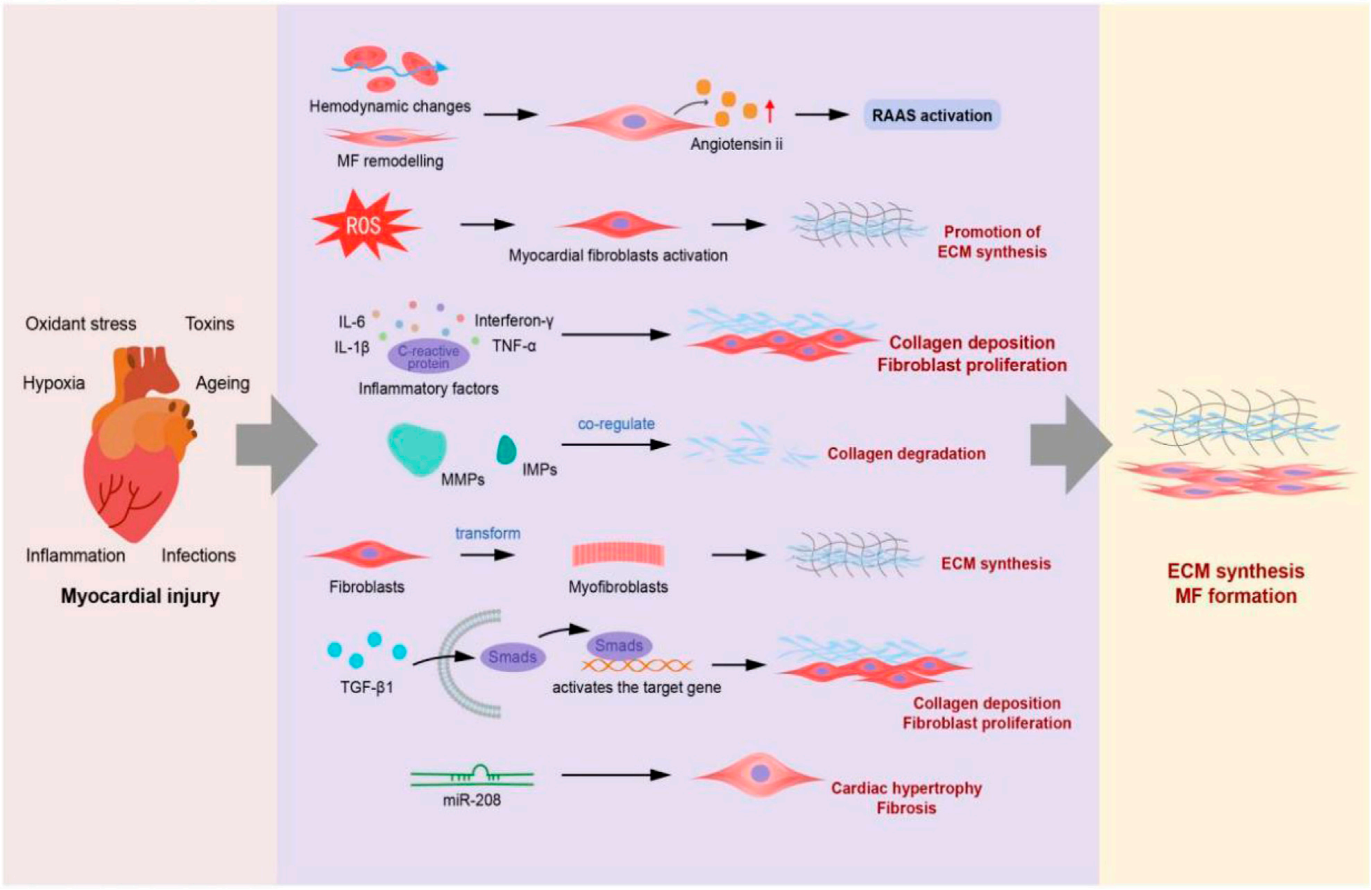
Mechanisms of myocardial fibrosis (The RAAS, oxidative stress, immune inflammatory mechanisms, matrix metalloproteinase system, fibroblast overproliferation, and TGF-β1/Smad3 signalling pathway lead to ECM deposition and sustained myocardial fibrosis).

The mechanism of MF is mutifactorial. Studies have shown that the occurrence and development of MF are closely related to the renin–angiotensin–aldosterone system (RAAS), oxidative stress, immune inflammation, matrix metalloproteinase system, fibroblast proliferation, and TGF-β1/Smad3 signalling pathway (Figure 1).

### Renin–Angiotensin–Aldosterone System

Ke et al. (2018) suggested that the RAAS is not only an important part of endocrine system but also the main response system of myocardial cells under the influence of pressure or capacity load. Myocardial injury causes hemodynamic changes, MF remodelling, increased myocardial hardness, and decreased compliance, stimulating endogenous angiotensin II (Ang II) secretion. A study reported (Goldsmith, 2004) that the decrease in heart contractility of patients with heart failure and renal blood flow activated the RAAS and increased the levels of many substances such as renin, Ang II, aldosterone (ALD), endothelin, antidiuretic hormone, and cytokines in blood and tissues. Among these molecules, Ang II and aldosterone are the main effector molecules that cause MF. These molecules participate in the formation of MF through various signalling pathways. ALD is a cardiovascular risk factor independent of Ang II that increases the concentration of reduced coenzyme II oxidase and superoxide anion, decreases the activity of nitric oxide (NO) in vascular endothelium, and regulates the inflammation of coronary artery and peripheral blood vessels, thereby serving as one of the important factors affecting MF development (Zhang et al., 2020).

### Oxidative Stress

Many studies have shown that oxidative stress affects fibrosis formation in the liver, lung, pulmonary artery, nervous system, and heart, thereby playing a crucial role in MF formation (Gutiérrez-Tenorio et al., 2017). On the one hand, the increase in reactive oxygen species after myocardial injury can directly activate myocardial fibroblasts, induce their differentiation and proliferation, promote ECM synthesis, and induce MF formation. On the other hand, it can indirectly participate in MF formation by mediating the influence of cytokines, growth factors, and Ang II on the function of fibroblasts and ECM metabolism (Xu et al., 2011). Moreover, the increased generation of molecules with high molecular activity such as reactive oxygen species (ROS) in the body disrupts the dynamic balance, resulting in their excessive accumulation. Oxidative stress finally causes damage to the lysosomes and mitochondria in myocardial cells, thus participating in the occurrence and progression of MF (Chaudhuri et al., 2019). Other ROS such as superoxide dismutase (SOD), glutathione catalase (GSH-Px), malondialdehyde (MDA), and other haemoglobin peroxidases defend free radical reactions at different levels.

### Inflammatory Reaction

The immune inflammatory reaction plays a crucial role in MF pathogenesis by producing inflammatory factors such as tumor necrosis factor α (TNF-α), interferon-γ, interleukin-6 (IL-6), interleukin -1β (IL-1β), C-reactive protein, macrophage chemotactic protein-1, and intercellular adhesion molecule. A study (Prabhu and Frangogiannis, 2016) indicated that the inflammatory factors can promote the expression of fibroblasts, alter myocardial interstitial components, and promote the migration of fibroblasts, whereas oxygen free radicals induce MF in many ways. The enhanced expression and activity of inflammatory cells and inflammatory factors results in the increased static fibroblast proliferation and differentiation into myofibroblasts and collagen deposition, causing MF.

### Matrix Metalloproteinase System Imbalance

MF is characterized by excessive deposition of collagen fibres in the myocardium, which is caused particularly by an imbalance in the synthesis and degradation of collagen fibres. Collagen degradation is regulated by extracellular matrix metalloproteinases (MMPs) and IMPs. MMP is a zinc-dependent endopeptidase and the main enzyme that controls the ECM balance, as well as the factor for cardiac remodelling after myocardial infarction (Fan et al., 2014). Tissue inhibitor of MMP-1 (TIMP-1) is a type of glycoprotein that is present in body fluids and tissues, it can inhibit the activity of almost all MMPs, particularly of MMP-1, MMP-3, and MMP-9 (Wang et al., 2015). TIMP-1 and MMPs play a common role in maintaining normal myocardial structure and function in the collagen degradation system. The change in the TIMP-1/MMP ratio leads to an imbalance in collagen synthesis and degradation, leading to MF.

### Activation of Fibroblasts

Cardiac fibroblasts, which originate from the epicardial endothelium of embryo, control various properties and forms of myocardial injury reactions in the heart (Fernández-Ruiz, 2021). After myocardial system injury, fibroblasts are transformed into myofibroblasts due to the interaction among various acute inflammatory cell transforming growth factors and cytokines, thereby promoting MF. Presently, vimentin is widely used as the extracellular matrix of myofibroblasts, whereas α-smooth muscle actin is used as a specific marker (Baiker et al., 2018) to promote the activation of myofibroblasts. The trans-differentiation of myofibroblasts requires several key factors. First, TGF-β is activated in the cardiomyocytes located in the interstitial of myocardial fibroblasts and smad3 signal transduction pathway through integrin (Rocher et al., 2020). Second, the changes in the response to mechanical stress of myocardial cells or regulation of growth factor signal transmission through integrin promotes the trans-differentiation of myofibroblasts. Third, integrin and synthetase promote the synthesis and expression of extracellular matrix surface receptors, which may play a crucial role in signal transduction mediated by cardiac fibroblasts (Hinz, 2010). In the pathogenesis of CFs activation and cardiac fibrosis, it has been observed and recorded that the endogenous component is the modification in the matrix structure or composition, the induction of multiple growth factors and cytokines [such as TGF-β, connective tissue growth factor (CTGF), platelet-derived growth factor (PDGF), Ang II, endothelin-1], the increase of mechanical stress, the upregulation of inflammatory signaling and chemokines [such as TNF-α, IL-1β, IL-6, C-C motif chemokine ligand 2 (CCL2)] (Frangogiannis, 2004; Kong et al., 2014; Schaefer, 2018). Furthermore, some commonly used clinical drugs, such as Doxorubicin (Levick et al., 2019) and Cyclophosphamide (Ayza et al., 2020), can also cause CFS activation and myocardial fibrosis.

### TGF-β1/Smad Signalling Pathway

TGF-β1 is a typical cell growth factor that promotes fibrosis (Meng et al., 2016). It can regulate cell growth and differentiation, promote cell proliferation. Additionally, it plays an important role in MF formation. *In vivo* experiments have demonstrated that TGF-β1 plays a vital role in cardiac fibrosis development (Meyer et al., 2012; Livingston et al., 2013). Moreover, *in vitro* experiments revealed that TGF-β1 could induce fibroblast proliferation and promote fibrosis (Strutz et al., 2001). Smad is an important intracellular protein molecule involved in the downstream pathway of the TGF-β1 signal transduction system.TGF-β1 binds to a type II receptor (TβRII), which induces TβRII to phosphorylate and recruit the type I receptor (TβRI). These events can trigg er phosphorylation of receptor-regulated Smads (R-Smads) and heterodimerization with co-chaperones. Next, the het erodimer complex enters the nucleus to regulate the expression of target genes via a combination with the SMAD-binding element (Massagu´e, 2014; Ricard-Blum et al., 2018). There is substantial evidence that TGF-β can both stimulate fibroblast-to-myofibroblast trans-differentiation and inhibit ECM degradation (Biernacka and Frangogiannis, 2011; Jiang et al., 2014). Therefore, targeting TGF-β is an effective strategy to reduce CFs activation and fibrosis.

### micRNA

Studies have also reported the role of micRNAs in the occurrence and development of MF. micRNAs are a large class of non-coding single-stranded small RNAs (Bayoumi et al., 2017), and the role of micRNAs in cardiac remodelling and fibrosis development has been widely studied (Zarkesh et al., 2018). For example, recent studies have shown that the silencing or inhibition of miR-21 (Thum et al., 2008) and miR-34 (Huang et al., 2014) can alleviate cardiac fibrosis. MiR-378 (Nagalingam et al., 2014), miR-22 (Yousefi et al., 2020), MiR-29 (Van Rooij et al., 2008) deficiency or downregulation can induce the expression of fibrosis genes, promote the production of collagen and procollagen, and induce MF. These data indicate that multiple micRNAs may be involved in promoting fibrosis. Another study (Thum et al., 2008) reported that the enhanced expression of miR-2 could inhibit the ERK–MAPK signalling pathway inhibitor SPRY1, thereby enhancing the activity of this signalling pathway of cardiac fibroblasts, promoting the survival of fibroblasts, and secreting growth factors. In addition, micRNAs such as miR-29, miR-1, and miR-133 play different roles in the development of myocardial fibrosis (Tarbit et al., 2019).

## Related Research on the Prevention and Treatment of Myocardial Fibrosis Based on Traditional Chinese Medicine

Although scholars have put forward various theories to explain the mechanism of MF, the exact mechanism remains unknown. Therefore, it is of great practical significance to discuss MF pathogenesis, and the development of traditional Chinese medicine has formed a unique style after several centuries. Traditional Chinese medicine generally includes the active ingredients of Chinese medicine, Chinese medicine decoction and Chinese patent medicine, which is an excellent source of anti-fibrosis drugs. Related literature published in recent years has revealed the mechanism of action of traditional Chinese medicine in alleviating MF, which is summarized in Table 1.

**TABLE 1:** Key areas and mechanisms of traditional Chinese medicine in treating myocardial fibrosis.

Action	Related Molecular mechanism	Related Pharmacological Indicators	References
Affecting RAAS	Inhibition of the activation of RAAS.ACE/AngII/AT1R-TGF-β1axis. AngII-NADP oxidation -ROS-MMPs pathway	Ang II, ACE, AT1R, AT2R, Ang I, ALD, Renin, CTGF, TGF-β1	Pang et al., (2015); Lu et al., (2015); Xu et al., (2015b); Ma, (2015); Duan and Yao, (2019)
Inhibition of fibroblast activation	Regulation of TGF-β1-Smad/TAK1/AKT/MAPK signalling pathway. Blocking cell cycle	TGF-β1, CTGF, JNK, P38, ERK, TLR4, Smad2, Smad3	Che et al., (2019); Jiang et al., (2020); Hu et al., (2021); Zhang et al., (2017); Ge et al., (2018); Tan et al., (2020); Shen et al., (2017); Wu et al., (2021); Ding et al., (2020)
Regulation of TGF-β1/Smad	Down regulation of TGF-β1 and regulation of Smad family protein. Inhibition of cardiac collagen proliferation	TGF-β1, Smad1, Smad2, Smad3, Smad4, Smad7, collagen I, III, (PAI)-1	Shen et al., (2014); Shen and Wu, (2016); Zhao et al., (2016); Chen et al., (2017); Zhao et al., (2020a); Hong et al., (2010); Wang et al., (2016a); Li and Ning, (2020); Shen et al., (2012); Zhan et al., (2014); Ma et al., (2016)
Oxidative stress	Reduction in the content of ROS and MDA. Increase in the contents of HO-1, GSH-PX, SOD, and NOX2. Activation of Nrf2/ARE signal pathway	ROS, MDA, HO-1, GSH-PX, SOD, NOX2, NADP/NADPH	Liu et al., (2014); Uzayisenga et al., (2014); Jia et al., (2018); Zhang et al., (2011); Fan et al., (2013); Wang et al., (2019); Zhao et al., (2020b)
Inhibition of extracellular matrix remodelling	Adjusting the metabolism of ECM and the balance of MMPs/TIMPs. EndoMT suppression	MMP-2, MMP-9, TIMP1, TIMP-2, NOX2, Collagen Ⅰ, Collagen Ⅲ	Li et al., (2018); Yu et al., (2016); Zhao et al., (2018); Gao and Zhu, (2009); Li et al., (2010); Ma et al., (2012); Qi et al., (2018); Fu et al., (2018); Qin et al., (2019)
Anti- inflammatory reaction	Inhibition of TNF-α/NF-κB, IL-6/STAT3, and TLR4/TAK1/NF-κB signalling pathway. Inhibition of the content of various inflammatory factors.	TNF-α, IL-6, IL-12, IFN-r, IL-18, IL-4, IL-5, TGF-β1, CTGF, IL-β, hs-CRP, NLRP inflammatory corpuscles	Yang et al., (2010); Zhao et al., (2010); Li et al., (2014); Yang et al., (2014); Kang et al., (2016); Zhang and Zhi, (2014); Yang et al., (2018); Lu et al., (2019); Zhang et al., (2013); Sun et al., (2021)
Regulate micRNA	Regulate cross-talking of miRNAs.	miR-15b, miR-133a, miR-29b, microRNA-200a, miRNA-1, miRNA-13, miRNA-1/miR-21, miR-22, miR-34a, miR-181a, miR-17	Liu et al., (2018b); Ma et al., (2020); Zheng et al., (2020); Qi, (2018); Chen, (2019); Liu et al., (2019); Zhang et al., (2019); Zhu et al., (2019); Wang et al., (2020); Wei et al., (2020)

### Regulation of the Renin–Angiotensin–Aldosterone System

It is known that the active ingredients and compounds of TCM can inhibit myocardial fibrosis by regulating RAAS. Curcumin is a natural polyphenol and yellow pigment extracted by enzyme method from turmeric that exhibits strong antioxidant and anti-inflammatory effects (Kotha and Luthria, 2019). Emerging evidence suggests that curcumin can be used to prevent and treat MF when pathological fibrotic changes occur in the myocardium in vivio. Curcumin (150 mg/kg/day) can antagonise the angiotensin-converting enzyme (ACE) and ATI receptor, increase the expression of AT2 receptor, and reduce the expression of ACE and ATI receptor and myocardial collagen accumulation, eventually improving the cardiac function of rats with myocardial infarction and reducing the degree of fibrosis (Pang et al., 2015). Qisheng Yiqi Dripping Pills (QSYQDP), a representative Chinese patent medicine composed of *Radix Astragali, Radix Salviae Miltiorrhizae, Radix Notoginseng* and *Lignum Dalbergiae Odoriferae*. QSYQDP is approved by the China State Food and Drug Administration in 2003 for the treatment of cardiovascular disease. Numerous pharmacological studies have revealed the synergistic effect of QSYQDP on cardiovascular diseases (Chang et al., 2019). Recently, QSYQDP (175 mg/kg/day) (Lu et al., 2015) has been shown to alleviate cardiac fibrosis by blocking the activation of RAAS and RAAS activation pathways, particularly by regulating the expression of AT1R and AT2R, and restoring the Ang II-NADPH oxidation-ROS-MMP pathway. This finding indicates that QSYQDP is a promising traditional Chinese medicine for treating MF. Luhong Granules (LHG) are mainly composed of *Antlers, Carthamus Tinctorius L, Psoralea Corylifolia Linn, Epimedium, Cornus Officinalis, Fructus Ligustri Lucidi* and *Aquilaria Sinensis (Lour.) Gilg,* which is prepared in the ratio of 15: 9: 30: 20: 15: 30: 6 (Xu et al., 2015a). According to literature, LHG can reduce plasma Ang II and aldosterone (ALD) in rats with heart failure and thus improve their cardiac function (Xu et al., 2015a). Studies have proved that LHG (1.25 g/kg/day) (Xu et al., 2015b) can activate the ACE2-Ang (1–7) axis, decrease the expression level of Ang II, and inhibit MF in rats with heart failure after myocardial infarction. Besides, the main ingredients of LHG need to be identifified and are worthy of further study in MF treatment. Luqi Recipe (LQR) is mainly composed of *Radix Astragali, Codonopsis Pilosula, Carthamus Tinctorius L, Cinnamomum cassia Presl, Antler Powder,* which is prepared at a ratio of 30: 15: 9: 15: 9: 2 (Liu et al., 2018a). It is proved that LQR (14.56 g/kg/day) (Liu et al., 2018a) can significantly reduce the levels of Ang I, Ang II, and ALD in rats with hypertension and heart failure and reduce the content of myocardial collagen, suggesting that LQR can inhibit the RAAS and alleviate MF to a certain extent. However, the main ingredients of LQR was not provided in the article. Matrine is an alkaloid extracted from the dried roots, plants, and fruits of *Sophora flavescens* belonging to the family Leguminosae. It has antioxidant and anti-myocardial fibrosis effects. A study reported that matrine (0.25 mmol/L, 0.5 mmol/L, 1.0 mmol/L) (Dai et al., 2013) can significantly reduce the contents of Ang II type 1 receptor (AT1R) and CTGF in CCFs induced by Ang II, thus inhibiting the proliferation of myocardial cells and promoting collagen synthesis. Kangxin Shuai Granule (KXSG) is composed of Chinese herbal medicines such as *Radix Astragali, Cornus Officinalis, Typha Angustifolia*, and *Sargassum Seaweed* and has the functions of invigorating qi, eliminating phlegm, and inducing diuresis. The efficacy of this granule in promoting blood circulation and resolving hard mass (Fang et al., 2009) indicates its preventive and therapeutic effects on chronic heart failure and myocardial fibrosis (Jie et al., 2015). Furthermore, another study found that KXSG (24.14 g/kg, 48.29 g/kg) (Ma, 2015) could significantly reduce the plasma aldosterone and renin activity and the TGF-β content in the cardiomyocytes of rats with diastolic heart failure and thus play a role in alleviating MF by effectively inhibiting RAAS overactivation. However, the ratio of the compound was not provided in the article. Qiangxin Capsule (QXC) is mainly composed of *Yuanhu*, *Aconitum Carmichaeli Debx, Panax Notoginseng, Radix Ginseng Rubra, Salvia miltiorrhiza, Radix Astragali,* Tinglizi, *Poria Cocos, Radix Paeoniae Alba* and *Atractylodes Macrocephala Koidz* at a ratio of 1.5: 1.5: 1: 2: 2: 5: 3: 2: 1.5: 2 (Duan and Yao, 2019). A study proved that (Duan and Yao, 2019) the QXC (10 mg/kg) can clearly reduce the left ventricle and heart mass index and the serum contents of ALD, renin, TGF-β, and Ang II in rats. The results showed that QXC could inhibit the left ventricular remodelling after AMI in rats, significantly improve the cardiac function of rats, and reduce the degree of MF by inhibiting RAAS activation.

### Inhibition of Fibroblast Proliferation

Berberine is a quaternary ammonium alkaloid isolated from natural drug *Coptis Chinensis* by solvent extraction (Chen et al., 2008). It is yellow needle crystal and tastes bitter. It has antimicrobial, antifungal, and anti-inflammatory properties. Some scholars have confirmed through *in vivo* and *in vitro* experiments (Che et al., 2019) that berberine can reduce the infiltration of macrophages into the rat myocardium stimulated by isoproterenol and inhibit the TGF-β1/smads signaling pathway to prevent fibroblasts from transforming into activated secretory myofibroblasts, thereby protecting the heart from injury. *In vivo* experiments have demonstrated that (Jiang et al., 2020) berberine (10 mg/kg/day, 60 mg/kg/day) can reduce the cardiac insufficiency and MF induced by Ang II in rats in a concentration-dependent manner and decreased the phosphorylation level of CDK2-T160 in the ventricular tissue. *In vitro* experiments demonstrated that berberine (20 μmol/L, 200 μmol/L) inhibited the proliferation of cardiac fibroblasts induced by Ang II in a concentration-dependent manner, arrested the cell cycle at the G0/G1 phase, and reduced the phosphorylation level of CDK2-T160 fibroblasts. The findings indicated that berberine can significantly inhibit MF and proliferation of cardiac fibroblasts and protected the cardiac function through CDK2-T160 activity dependence. *Ginkgo Biloba* extract (GBE) is an effective component that is extracted from *G. biloba* leaves by alcohol solution. Studies havereported that GBE (Mesquia et al., 2017) has an anti-inflammatory effect and plays an important role in lowering blood lipid, dilating blood vessels, scavenging free radicals, and protecting against cardiovascular and cerebrovascular diseases. Recent studies have reported that a high concentration of GBE (20 ng/ml) can effectively inhibit the growth of CFb in SD suckling mice induced by TGF-β1, and its mechanism may be related to the downregulation of CTGF, JNK, and P38 expressions in the TGF-β1-Smad/TAK1 signalling pathway (Hu et al., 2021). Oxymatrine (OMT) is a natural alkaloid that is mainly extracted from *S. flavescens* (Leguminosae, family), which has anti-inflammatory, antiarrhythmic, and anti-fibrotic pharmacological effects (Yang et al., 2019).Zhang et al. (2017) reported that the administration of 50 and 100 mg/L oxymatrine 2 h in advance could prevent the decline in the survival rate of isolated rat cardiomyocytes induced byTGF-β1, the survival rate increased from 85.25% to 94.27% and 98.53% in the control group. OMT can inhibit the TGF-β1/MAPK signalling pathway and protect cardiomyocytes by downregulating the protein expression of phosphorylated p38, phosphorylated JNK, and phosphorylated ERK1/2 induced by TGF-β 1. *Lonicera japonica* flavonoids are effective components extracted from natural drug *Lonicera japonica* by alcohol method*.* A study (Ge et al., 2018) demonstrated that the flavonoid extracts from *Lonicera japonica* (200 μg/ml, 400 μg/ml, 600 μg/ml) could significantly increase the percentage of G0/G1 phase cells of cardiac fibroblasts, decrease the percentage of S phase and G2/M phase cells, and block the cardiac fibroblast cells. Flavonoid extracts from *L. japonica* Thunb could remarkably inhibit cardiac fibroblast proliferation induced by Ang II in a dose-dependent manner. The mechanism could be related to the inhibition of cardiac fibroblast proliferation and blocking of cardiac fibroblast cell cycle. Honokiol (HKL), extracted from *Magnolia officinalis*, is a diphenol compound havingpharmacological effects such as anti-tumour, antibacterial, anti-inflammatory, and antioxidation and is often used to treat arrhythmia and cardiocerebral ischaemia/reperfusion injury (Zhang et al., 2018). Tan et al. (2020) studied the effect of HKL on themigration of cardiac fibroblasts stimulated by TGF-*β*1 and found that HKL (2.5 μ mol/L) could reduce the proliferation and migration of cardiac fibroblasts induced by TGF-*β*1 in neonatal rats. Inhibition of the deposition of Fibronectin (FN) protein can alleviate MF. Shensong Yangxin Powder (SSYXP) is a traditional Chinese medicine preparation, including 12 components: *Panax Ginseng*, *Panax Ginseng Root*, *Cornus Officinalis, Salvia Japonica Thunb, semen, Viscum Coloratum, Red Paeonia suffruticosa, Tubiechong, Nardostachys Jatamansi DC, Berberine, Schisandra Sphenanthera Rehd. et Wils* and *Longgu.* It is usually used to treat arrhythmia and myocardial fibrosis (Chen et al., 2016).Shen et al. (2017) demonstrated that SSYXP (100 μg/ml) could protect the proliferation, differentiation, and activation of cardiac fibroblasts induced by TGF-β1. It plays an anti-fibrosis role mainly by inhibiting the Akt pathway. Wu et al. (2021) found that Curcumin (25 μmol/L) can inhibit the growth and proliferation of CFs induced by Ang II, downregulate the expression of TLR4 and TGF-β, and upregulate the expression of BAMBI, which provides strong evidence for the discovery of anti-fibrosis drugs. Resveratrol (RES) is a natural polyphenol compound with strong antioxidant and anti-inflammatory activities, which mainly present in grapes and peanuts. Studies have reported that RES can interfere with the activation of fibroblasts and play a therapeutic role in cardiac hypertrophy, MF, and heart failure (Qin et al., 2012).Ding et al. (2020) confirmed that the anti-myocardial-fibrosis effect of RES (25 μmol/L) may be related to inhibition of the proliferation and differentiation of cardiac fibroblasts induced by Ang II and downregulation of the TGF-β1/Smads signalling pathway.

### Downregulation of TGF-β1 Expression and Regulation of the TGF-β1/Smad Signalling Pathway

Buyang Huanwu Decoction (BYHWD) is mainly composed of *Radix Astragali, Angelica Sinensis Tail, Radix Paeoniae Rubra, Pheretima, Ligusticum Chuan Xiong, Carthamus Tinctorius L, Taoren* at a ratio of 40: 2: 2: 1.7: 1: 1: 1*,* which is a classic prescription drug for invigorating qi and removing blood stasis. Studies have confirmed that BYHWD exerts protective effects on myocardial ischaemia injury (Wang et al., 2011). Chen et al. (2017) observed the effect of BYHWD on rats with hypertensive heart failure simulated by pressure overload. The results showed that BYHWD (2.0 g/kg/day) can significantly downregulateTGF-β1, inhibit phosphorylation of MAPKs and Smad3, and protect the heart by reducing the expression of the pro-inflammatory cytokines IL-6 and TNF-α and inhibiting the inflammatory reaction against MF. Shengmai Powder (SMP) is mainly composed of *Panax Ginseng, Ophiopogon Japonicus, Schisandra Chinensis* at a ratio of 9: 9: 6, which is used to treat chronic heart failure and myocardial fibrosis. Zhao et al. (2016) administered SMP to type 2 diabetic mice for 24 weeks and found a decrease in TGF-β1 expression and Smad2/3 Phosphorylation, the downregulation of MMP-2 and MMP-9 expression could significantly improve the left ventricular diastolic function and the degree of MF. Another *in vivo* experimental study reported that (Zhao et al., 2020a) SMP (5.0 g/kg/day) can significantly improve the cardiac function of ISO-induced heart failure rats, and its mechanism may be related to the TGF-β1/Smad3 pathway regulation. In addition, Shensong Yangxin Capsule (SSYXC) can not only prevent myocardial hypertrophy caused by pressure overload but also improve the degree of MF in diabetic cardiomyopathy (Shen et al., 2014; Shen and Wu, 2016) by inhibiting the TGF-β1/Smad signal transduction pathway. Furthermore, modern pharmacological research has revealed that Tongxinluo Capsule (TXLC) can significantly improve coronary microcirculation, reduce inflammation and oxidation reactions,and directlyor indirectly protect the cardiomyocytes (Cui, 2014). The main components of TXLC are *Ginseng*, *Quanxie*, *Centipede*, *Leech, Soil insects, Cicada skin, Red peony, Borneolum, Boswellia carteriiBirdw(Ruxiang), Dalbergia odorifera (Jiangxiang), Santalum album L.* and *Semen Ziziphi Spinosae.* Wang et al. (2016a) investigated the effect of TXLC on MF in diabetic rats and found that it could downregulate the protein and mRNA levels of TGF-β1 and Smad3, upregulate Smad7 expression, and prevent Smad2/3 phosphorylation and TGF-β1 intracellular signal transduction.Hong et al. (2010) established a MF model after myocardial infarction in rats by ligating the anterior descending branch of coronary artery, TXLC (1 g/kg/day) significantly improve the cardiac function of rats with myocardial infarctionby inhibiting the TGF-β1/Smad signalling pathway and thus reducing cardiac collagen deposition and inhibiting MF and left ventricular remodelling. *Salvia Miltiorrhiza* can clears heat and exertsa tranquilising effect on mind, thereby relieving pain during menstruation, promoting blood circulation, and removing blood stasis. The active components of this medicine are the drugs that are commonly used for treating cardiovascular diseases such as danshensu (Wang et al., 2016b) and Tanshinone IIA (Chen et al., 2015). Danshensu (10 mg/kg, 20 mg/kg, 40 mg/kg) (Li and Ning, 2020) can effectively improve the degree of MF in rats with acute myocardial infarction by dose dependentlyinhibiting the expression of TGF-β and CTGF and regulating the TGF-β1/Smadsignalling pathway. Tanshinone ⅡA (70 mg/kg/day) can downregulate the TGF-β1/Smad2/3 signalling pathway activity in rats with MF induced by Ang II. Moreover, it can effectively inhibit collagen deposition, overexpression of CTGF, and plasminogen activator inhibitor (PAI)-1 and alleviate MF in rats (Zhan et al., 2014; Ma et al., 2016).Shen et al. (2012) studied the protective effect of OMT on experimentally induced MF in rats with AMI and confirmed that OMT (50 mg/kg) could inhibit the TGF-β1-Smads signalling pathway, inhibit the upregulation of TGF-β1, Smad2, Smad3, Smad4 mRNA expressions and increase the Level of Smad7 mRNA, thereby alleviating MF inrats with AMI.

### Regulation of Free Radical Metabolism *In Vivo*

TCMs and its active ingredients can treat myocardial fibrosis through regulate free radical metabolism. Paeonol (Pae), also known as paeonol, is the major component of the root bark of *Ranunculaceae peony* and the dried root or whole plant of Ramat Xu Changqing, which has anti-inflammatory and antioxidant effects (Liu et al., 2013). Ppanax notoginseng saponins (PNS) are the main effective components of *Panax Notoginseng* from Araliaceae that are mostly used to prevent and treat cardiovascular and cerebrovascular diseases. These compounds exhibit anti-cancer, neuroprotective, and hypoglycaemic properties (Uzayisenga et al., 2014). Pae (80 mg/kg) in combination with PNS (100 mg/kg) (Jia et al., 2018) was found to upregulate HO-1 and SOD expression in the myocardial tissue of a diabetic cardiomyopathy model induced by high fat and high sugar feeding combined with intraperitoneal injection of streptozotocin, as well as reduce the level of MDA and ROS. Furthermore, it can inhibit the expression of type I and type III collagen and activate the Nrf2/ARE signallingpathway through anti-oxidative stress, which can synergistically alleviate MF caused by DCM.Liu et al. (2014) reported that curcumin (200 mg/kg) could significantly improve the weight loss and fasting blood glucose in DCM rats, inhibit MDA production in myocardium, and increase glutathione peroxidase activity. Reduced release of serum cardiac troponin I and decreased expression of protein kinase C in the myocardial tissue indicated that the protective effect of curcumin on DCM in rats may be related to the inhibition of oxidative stress. The experimental results of Hu et al. (2015) indicated that hesperetin (100 μmol/L) may inhibit the proliferation of cardiac fibroblasts and effectively inhibit the occurrence and development of MF by directly inhibiting ROS production. Total flavonoids of *Sophora flavescens* are the most bioactive compounds extracted from *S. flavescens* by alcohol method that exhibit a wide range of physiological activities, including antibacterial, antioxidant, and anti-inflammatory (Zhang et al., 2011; Chong et al., 2013). A study (Fan et al., 2013) have confirmed that total flavonoids of *Sophora flavescens* (200 mg/kg, 400 mg/kg) antagonises Ang II generation in the myocardium by increasing the NO concentration in local tissues of the myocardium or blood vessels. Reduce MDA content, increase myocardial SOD activity to resist lipid peroxidation and inhibit collagen synthesis to resist MF in rats. Some Compounds of TCMs are known to regulate free radical metabolism. Tetramethylpyrazine (TMP) is a biomonomer with molecular formula C_8_H_12_N_2_·HCl·_2_H_2_O extracted from the traditional Chinese herbal medicine Chuanxiong. An experimental study on Tetramethylpyrazine Phosphate Tablets (TPT) in rats with isoproterenol-induced heart failure confirmed that TPT (4 mg/kg) could increase the activities of SOD and GSH-Px and decrease the level of MDA, thereby reducing oxidative stress, improving cardiac function, and inhibiting MF (Guo et al., 2012). QSYQDP is listed as a Chinese herb preparation, which is widely used in the treatment of coronary heart disease and heart failure (Wang et al., 2019).Shu et al. (2012) confirmed that QSYQDP (250 mg/kg/day) can increase the circulating SOD and GSH-PX levels, inhibit the production of ROS, and thus inhibit MF development. Simiao Yongan Decoction (SMYAD) is aopular formula of Chinese herbal medicine that exerts the effects such as clearing away heat and toxic materials, promoting blood circulation, and removing blood stasis. SMYAD is mainly composed of *Lonicerae Japonicae Flos, Radix Scrophulariae, Radix Angelica Sinensis, Radix Glycyrrhizae* at ratio of 3: 3: 2: 1*.* Numerous pharmacological studies have shown that it functions asan antioxidant and also functions in lowering the blood lipid level and reducing the atherosclerotic plaque (Zhao et al., 2020b). A study have revealed that the mechanism of action of SMYAD in the treatment of MF is related to its antioxidant effect. After ISO injury, collagen deposition in the myocardium of the mice treated with SMYAD decreased significantly, which may be attributed to ROS and SOD removal, NOX2 balance restoration, and NADP/NADPH ratio reduction by SMYAD (Ren et al., 2019).

### Inhibition of Extracellular Matrix Remodelling

Animal experiments (Li et al., 2018) have shown that berberine can downregulate theexpression of insulin-like growth factor-1 receptor (IGF-1R) in the heart of diabetic rats and then reduce the expression of MMP-2/MMP-9, α-SMA, and type-Icollagen in the heart to alleviate MF and restoring heart dysfunction, thus playing a cardioprotective role in diabetic rats. Shenzhu Xinkang Decoction (SZXKD) is mainly composed of *Codonopsis Pilosula, Radix Ophiopogonis, Radix Polygonatum Odoratum, Radix Astragali, Schisandra Chinensis, Salviae Miltiorrhizae* at ratio of 20: 15: 10: 20: 6: 20, which is a representative prescription for the treatment of chronic heart failure and fibrosis (Yu et al., 2013). Yu et al. (2016) confirmed that SZXKD high dose group (44 g/kg/day) can reduce MMP9 expression, increase TIMP1 expression, and improve MF in rats with chronic heart failure. A study by Zhao et al. (2018) have reported that QSYQDP (270 mg/kg/day) can improve the cardiac systolic and diastolic functions and exerts anti-fibrosis effects by inhibiting collagen deposition, MMTO expression, and CF proliferation and differentiation in rats with MF following myocardial infarction. Fuzheng Huayu Capsule (FZHYC) is an FDA-approved drug in China that is used to treat organ fibrosis, consists of six Chinese herbs, including Semen Persicae (Tao-ren), Cordyceps Sinensis, Gynostemma Pentaphyllammak (Jiao-gulan), Radix Salvia Miltiorrhizae, Pollen Pini and Schisandrae Chinensis. It can promote blood circulation, remove blood stasis, and soften and resolve hard masses. Moreover, it exerts a strong anti-hepatic fibrosis effect (Liu et al., 2009; Song et al., 2017). In a study, FZHYC was found to improve not only liver fibrosis but also MF following myocardial infarction (Guo et al., 2020). Qi et al. (2018) confirmed that the FZHYC (0.4 g/kg/day) improves the degree of MF after myocardial infarction in rats by regulating MMP2, MMP9, TIMP1, and TIMP2 expressions and controlling the MMP2/TIMP2 and MMP9/TIMP1 balance. Li et al. (2010) studied the effect of Tanshinone IIA on spontaneously hypertensive rats (SHR) and confirmed that Tanshinone IIA (40 mg/kg) can reduce not only blood pressure but also ventricular mass, ventricular mass index, and myocardial collagen volume fraction (CVF) to different degrees. The mechanism of action of Tanshinone IIA is related to the inhibition of MMP-2, MMP-9, and TIMP-2 expressions and improvement in the MMP/TIMP ratio. Gao et al. (Gao and Zhu, 2009) reported that RES can effectively improve the imbalance of MMP-2/TIMP-2 expression and prevent atherosclerosis MF. Additionally, resveratrol caninterfere with MF induced by alcohol by inhibiting MMP-2 and MMP-9 expressions (Ma et al., 2012). Icarisid Ⅱ (ICS Ⅱ), a polyhydroxy flavonoid monomer component extracted from *Epimedium Brevicornum*, exerts anti-inflammatory and antioxidant effects and improves myocardial cell apoptosis (Khan et al., 2015; Wu et al., 2017).Fu et al. (2018) investigated the effect of ICS II on MF in Spontaneously Hypertensive Rats (SHR), the ICS II (8 mg/kg, 16 mg/kg) demonstrated significantly reduced collagen deposition, systolic blood pressure, left ventricular mass index, and MMP-2, MMP-9, collagen I, and collagen III expressions in the left ventricular Tissue. However, the expression of TIMP-1 was increased significantly. The findings indicated that ICS II can improve MF in SHR rats, and its mechanism may be related to lowering blood pressure, downregulating MMP-2 and MMP-9 expressions, and upregulating TIMP-1 expression. BYHWD (36 g/kg/day) (Qin et al., 2019) was reported to prevent MF in mice with viral myocarditis by inhibiting the proliferation of myocardial collagen, regulating MMP and TIMP expressions, and improving the MMP and TIMP imbalance.

### Inhibition of Inflammatory Reaction

Studies have shown that sodium tanshinone IIA sulfonate (STS) (100 mg/kg/day) (Yang et al., 2014) can significantly reduce the TNF-α level in plasma and NF-κB and TNF-α mRNA expressions in the myocardium of type 2 diabetic rats, which playing a role in myocardial protection by inhibiting the NF-κB inflammatory signalling pathway. The compound Qishen Yiqi can protect the myocardium by inhibiting the TNF-α/NF-κB and IL-6/STAT3 signalling pathways and slowing down the ventricular remodelling and MF caused by inflammation (Li et al., 2014). Tingli Shengmai Prescription (TLSMP) is composed of *Tinglizi, Radix Astragali, Red Ginseng Rubra,* Fructus Schisandrae Chinensis, Radix Salvia Miltiorrhizae, which is effective in treating chronic heart failure caused by qi deficiency and blood stasis, yang deficiency, and water stagnation. (TLSMP) (Zhao et al., 2010) candecrease the levels of inflammatory factors TNF-α and IL-6 in blood of rats with chronic heart failure and inhibit MF formation. Cinnamaldehyde is a compound separated from the cinnamon bark. Kang et al. (2016) reported that cinnamaldehyde (40 mg/kg, 80 mg/kg) can potentially alleviate heart inflammation and fibrosis by inhibiting NLRP inflammatory body activation and regulating the signal of TLR4/6-interleukin-1 mediator-related kinase (IRAK4)/1 mediated by CD36. Yang et al. (2010) treated MF in elderly hypertensive mice by administering the intraperitoneal injection of STS. The study confirmed that STS (100 mg/kg/day) can interfere with MF through inflammation and immune imbalance regulation by decreasing the Th1 factor (IL-12 and IFN-r) levels and increasing the TH2 factor (IL-4 and IL-5) levels in peripheral blood. Lingui Zhugan Decoction (LGZGD) is mainly composed of *Ting-lizi, Atractylodes Macrocephala Koidz, Poria Cocos(Schw.) Wolf,* Radix Salvia Miltiorrhizae, Cinnamomum Cassia Presl, Ze-lan, Radix Glycyrrhizae Preparata at a ratio of 30: 20: 15: 15: 10: 10: 3. It is widely used in the treatment of cardiovascular diseases and exhibits remarkable effects (Gu et al., 2020). Yang et al. (2018) reported that LGZGD can significantly reduce the serum IL-6, IL-18, and TNF-α levels in patients with CHF, inhibit the overexpression of inflammatory factors, and protect against MF. Radix *Astragalus Membranaceus* is a widely used traditional Chinese medicine for alleviating MF. Lu et al. (2019) studied the beneficial effect of *Astragalus* oral liquid on vascular inflammation in patients with chronic heart failure and MF. The IL-6, TNF-α, and hs-CRP levels in the observation group treated with *Astragalus* oral liquid (30 ml/day) were decreased compared with those in the control group. Therefore, *Astragalus* oral liquid could improve MF in chronic heart failure by inhibiting inflammatory factors.Zhang and Zhi (2014) analyzed the effect of *Astragalus* injection on serum TNF-α, IL-6, and Ang II levels in patients with chronic heart failure and reported that *Astragalus* injection (10 ml/day) can significantly reduce inflammatory factors, indicating that it exerts a protective effect on the myocardial cells. Triptolide (TP) is the main active natural product isolated from the medicinal plant *Tripterygium wilfordii* that plays a crucial role in resisting oxidation and inhibiting inflammation (Cui et al., 2017). In a rat abdominal aorta ligation model, TP was reported to reduce the release of inflammatory factors IL-1β and IL-6, reduce the activation of NF-κB, and reduce the release of inflammatory factors and production of TGF-β and Ang II by exerting an anti-myocardial fibrosis effect (Zhang et al., 2013). Another *in vivo* experiment indicated that (Sun et al., 2021) TP (100 μg/kg, 200 μg/kg) could reduce the release of TNF-α and IL-6 and theCK-MB and cTn-I contents in the blood of septic rats, thus significantly alleviating MF. The underlying mechanism may be related to the inhibition of the TLR4/TAK1/NF-κB inflammatory signalling pathway.

### Regulation of the micRNA

Based on the ancient Danggui Buxue Decoction, *Angelica sinensis* and *Hedysarum hedysarum* were used as raw materials to prepare the ultrafiltration of *Radix Angelica Sinensis* and *Radix Hedysari* (RAS-RH) (Ma et al., 2019). *In vivo* experiments revealed that RAS-RH (50 mg/kg/day) (Ma et al., 2020) can alleviate the MF induced by X-ray in rats, and its mechanism may be related to the inhibition of microRNA-200a expression by RAS-RH. Xinkang Granule is mainly composed of *White Ginseng*, *Astragalus Membranaceus*, *Bupleurum*, *Cimicifuga*, *Platycodon grandiflorum*, *Poria cocos, Coix Seed*, *Zingiber officinale peel*, *Areca catechuL. peel*, *Tangerine peel*, *Cinnamon Twig*, *Aconitum carmichaelii Debx*, *Amomum villosum* at a ratio of 10: 30: 5: 5: 5: 15: 30: 10: 10: 10: 6: 10: 6. Liu et al. (Liu et al., 2018b) performed experiments with adriamycin model CHF rats, which indicated that XKG (2 g/kg/d) could decrease the miRNA-21 signalling pathway activation, increase the expression level of PTEN, and improve cardiac compliance. Berberine can effectively inhibit left ventricular hypertrophy and MF caused by various factors and improve cardiac function (Ying et al., 2018). Zheng et al. (2020) studied the effect of berberine on the expression of miR-29b in the pressure overload hypertrophy myocardium and applied its intervention to treat MF. The study confirmed that berberine (100 mg/kg/day) can inhibit myocardial hypertrophy and fibrosis in the pressure overload myocardial hypertrophy model by upregulating miR-29b expression and downregulating the expression of its target gene. According to a study, *Carthamus Tinctorius L.* can improve the cardiac function and ventricular remodelling in patients with ischaemic cardiomyopathy and prevent MF development (Yu et al., 2019). The Tinglizi can inhibit myocardial hypertrophy and fibrosis and correct heart failure (Chen et al., 2019). Compatibility between the *Carthamus Tinctorius L.* and Tinglizi (CTL-TLZ) can promote blood circulation, remove blood stasis and greatly enhance the anti-MF effect (Wang et al., 2021). CTL-TLZ is mainly comosed of *Carthamus Tinctorius L.* and *Tinglizi* at a ratio of 3: 5*.* Wang et al. (2020) observed the inhibitory effect of CTL-TLZ in MF in mice with heart failure following myocardial infarction. The experimental results showed that compared with the model group, CTL-TLZ.

(2.0 mg/m L) can Upregulation of miRNA-22 expression and downregulation of TGFβ-1 expression in the myocardial tissue, can downregulate the expression of COL1A1, COL3A1, and TGFβ-1, upregulate the expression of miRNA-22 and inhibit the proliferation of fibroblasts and collagen synthesis. In addition, this herb can inhibit MF in mice with heart failure after myocardial infarction, and the underlying mechanism may be related to the regulation of miRNA-22/TGFβ-1 signalling pathway activation in fibroblasts. Besides, the main ingredients of CTL-TLZ need to be identifified and are worthy of further study in MF treatment. Zhu et al. (2019) observed the effects of Fuzheng Huayu Recipe (FZHZR) on the proliferation, apoptosis, and miR-29b-5p expression in cardiac fibroblasts induced by Ang II. The results showed that the mechanism of FZHZR (100 μg/mL) against MF may be related to the inhibition of cardiac fibroblast proliferation induced by Ang II, promotion of cardiac fibroblast apoptosis, and regulation of miR-29b-5p expression. *Astragalus membranaceus* is an herb with many pharmacological functions. As one of the active ingredients of *Astragalus membranaceus*, Astragaloside IV has been proved to inhibit myocardial fibrosis. Astragaloside IV (10 mg/kg/day) inhibit cardiac fibrosis by targeting miR-135a and activating the TGF-β/Smads pathway (Wei et al., 2020). Liu et al. (Liu et al., 2019) reported that XKG (1.2 g/mL/day) could regulate miRNA-1 and miRNA-133/caspase-3 expressions, inhibit myocardial apoptosis, and resist MF. Yao et al. (Zhang et al., 2019) reported that RSV (50 μmol/L) can inhibit the proliferation of cardiac fibroblasts induced by TGF-β, and its mechanism may be the downregulation of miR-34a, miR-181a, and miR-17 expressions. Qi et al. (Qi, 2018) reported that FZHYC (0.4 g/kg/day) alleviates MF following myocardial infarction in rats by upregulating the expression of miR-29 family members. Qili Qiangxin Capsule (QLQXC) is a standardized Chinese herbal extract prepared from 11 Chinese herbs, including *Astragalus Membranaceus*, *Panax Ginseng*, *Aconitum Carmichaeli Debx, Salvia Miltiorrhiza Bunge*, dry seeds of *Lepidium Apetalum Willd*. or *Descurainia Sophia (L.) Webb ex prantl*, *Alisma Orientalis*, *Polygonatum Odoratum*, dry twigs of *Cinnamomum Cassia Presl, Carthamus Tinctorius L*, *Periploca Sepium Bg*e, and dried ripe peel of *Citrus Reticulata Blanco*. Chen et al. (Chen, 2019) reported that QLQXC (0.32 g/kg/day) can inhibit left ventricular remodelling and improve MF in rats with myocardial infarction to different degrees, and the underlying mechanism is related to the upregulation of miR-133a expression.

To sum up, the above TCM research review is shown in Table 2.

**TABLE 2:** The mechanism of TCM in the treatment of myocardial fibrosis.

Type of TCM	TCM	Type of study	Mechanism of action	References
The bioactive ingredientsOf TCM	Curcumin	*In vitro* and *In vivo*	Antagonise the ACE and ATI receptor	Pang et al. (2015)
		*In vivo*	Inhibit the growth and proliferation of CFs	Wu et al. (2021)
		*In vivo*	Inhibit MDA production, and increase glutathione	Liu et al. (2014)
			Peroxidase activity	
	Matrine	*In vitro*	Reduce the contents of AT1Rand CTGF	Dai et al. (2013)
	Berberine	*In vitro* and *In vivo*	Inhibit proliferation of cardiac fibroblasts;	Che et al., (2019); Jiang et al., (2020)
		*In vivo*	Reduce the expression of MMP-2/MMP-9, α-SMA, and type-I collagen	Li et al. (2018)
		*In vivo*	Upregulating miR-29b expression	Zheng et al. (2020)
	Ginkgo Biloba extract	*In vivo*	Inhibit TGF-β1-Smad/TAK1 signalling pathway	Hu et al. (2021)
	Oxymatrine	*In vitro*	Inhibit TGF-β1/MAPK signalling pathway	Zhang et al. (2017)
		*In vivo*	Inhibit TGF-β1-Smads signalling pathway	Shen et al. (2012)
	Flavonoid extracts from *Lonicera japonica*	*In vitro*	Blocking of cardiac fibroblast cell cycle	Ge et al. (2018)
	Honokiol	*In vitro*	Reduce the proliferation and migration of cardiac fibroblasts	Chen et al. (2016)
	resveratrol	*In vitro*	Down regulation TGF-β1/Smads signalling pathway	Ding et al. (2020)
		*In vivo*	Improve the imbalance of MMP-2/TIMP-2 expression	Gao and Zhu, (2009)
		*In vitro*	Down-regulation of miR-34a, miR-181a, and miR-17 expressions	Zhang et al. (2019)
		*In vivo*	Inhibiting MMP-2 and MMP-9 expressions	Ma et al. (2012)
	Danshensu	*In vivo*	Regulating the TGF-β1/Smad signalling pathway	Li and Ning, (2020)
	Tanshinone ⅡA	*In vivo*	Down regulate the TGF-β1/Smad2/3 signalling pathway	Shen et al., (2012); Zhan et al., (2014); Ma et al., (2016)
		*In vivo*	inhibition of MMP-2, MMP-9, and TIMP-2 expressions	Li et al. (2010)
			And improvement in the MMP/TIMP ratio	
	Paeonol in combination with *P. Notoginseng* saponins	*In vivo*	Upregulate HO-1 and SOD expression,reduce the level of MDA and ROS	Jia et al. (2018)
	Hesperidin	*In vitro*	Inhibiting ROS production	Hu et al. (2015)
	Total flavonoids of *Sophora flavescens*	*In vivo*	Reduce MDA content and increase myocardial SOD activity	Fan et al. (2013)
	Icarisid Ⅱ	*In vivo*	lowering blood pressure, downregulating MMP-2 and MMP-9 expressions, and upregulating TIMP-1 expression	Fu et al. (2018)
	Sodium Tanshinone ⅡA Sulphonate	*In vivo*	Reduce the TNF-α level and NF-κB and TNF-α mRNA expressions; inhibiting the NF-κB inflammatory signalling pathway	Yang et al. (2014)
		*In vivo*	Decreasing IL-12 and IFN-r levels and increasing IL-4 and IL-5 levels	Yang et al. (2010)
	Cinnamaldehyde	*In vivo*	Inhibiting NLRP inflammatory body activation and regulating the signal of TLR4/6-interleukin-1 mediator-related kinase (IRAK4)/1	Kang et al. (2016)
	Triptolide	*In vivo*	Reduce the release of IL-1β and IL-6, reduce the activation of NF-κB	Wang et al. (2021)
		*In vivo*	Reduce the release of TNF-α and IL-6 and the CK-MB and cTn-I contents	Sun et al. (2021)
	*Radix Angelica Sinensis* and Radix Hedysari	*In vivo*	Inhibiting microrna-200a expressions	Ma et al. (2020)
	Astragaloside IV	*In vitro*	Targeting miR-135a and activating the TGF-β/Smads pathway	Wei et al. (2020)
Chinese medicine decoction	Luqi Recipe	*In vivo*	Inhibit the RAAS; reduce the levels of Ang I, Ang II, and ALD	Liu et al. (2018a)
	Buyang Huanwu Decoction	*In vivo*	Inhibit phosphorylation of MAPKs and TGF-β1/Smad3	Chen et al. (2017)
		*In vivo*	Regulating MMP and TIMP expressions, and improving the MMP and TIMP imbalance	Qin et al. (2019)
	Shengmai Powder	*In vivo*	Regulation of the TGF-β1/Smad3 pathway	Zhao et al., (2016); Zhao et al., (2020a)
	Simiao Yongan Decoction	*In vivo*	Remove ROS and SOD, restore NOX2 balance and reduce	Ren et al. (2019)
			NADP/NADP H ratio; scavenging free radical	
	Tingli Shengmai Prescription	*In vivo*	Decrease the levels of TNF-α and IL-6	Zhao et al. (2010)
	Linggui Zhugan Decoction	Clinical experiment	Reduce the serum IL-6, IL-18, and TNF-α levels	Yang et al. (2018)
	Shenxinkang Decoction	*In vivo*	Reduce MMP9 expression, increase TIMP1 expression	Song et al. (2017)
	CTL-TLZ	*In vivo*	Regulation of miRNA-22/TGFβ-1 signalling pathway	Wang et al. (2020)
	Fuzheng Huayu Recipe	*In vitro*	Regulation of miR-29b-5p expression.	Zhu et al. (2019)
Chinese patent medicine	Qisheng Yiqi Dripping Pills	*In vivo*	Restoring the Ang II-NADPH oxidation-ROS-MMP pathway;	Lu et al. (2015)
		*In vivo*	Increase the circulating SOD and GSH-PX levels, inhibit the production of ROS	Shu et al. (2012)
		*In vivo*	Inhibiting collagen deposition, MMTO expression	Zhao et al. (2018)
		*In vivo*	Inhibiting the TNF-α/NF-κB and IL-6/STAT3 signalling pathways	Li et al. (2014)
	Luhong Granules	*In vivo*	Activate the ACE2-Ang (1–7) axis	Xu et al. (2015b)
	Kangxin Shuai Granule	*In vivo*	Inhibiting RAAS overactivation	Ma, (2015)
	Qiangxin Capsule	*In vivo*	Reduce the contents of ALD, renin, TGF-β, and Ang II	Duan and Yao, (2019)
	Shensong Yangxin Powder	*In vitro*	Inhibiting the Akt pathway	Shen et al. (2017)
	Shensong Yangxin Capsule	*In vitro*	Inhibiting the TGF-β1/Smad signalling pathway	Shen et al., (2014); Shen and Wu, (2016)
	Tongxinluo Capsule	*In vitro*	Regulation of the TGF-β1/Smad3 pathway	Hong et al., (2010); Wang et al., (2016a)
	Ligustrazine phosphate tablets	*In vivo*	Increase the activities of SOD and GSH-Px and decrease the level of MDA	Guo et al. (2012)
	Fuzheng Huayu Capsule	*In vivo*	Regulating MMP2, MMP9, TIMP1, and TIMP2 expressions and controlling the MMP2/TIMP2 and MMP9/TIMP1 balance	Qi et al. (2018)
		*In vivo*	Upregulating the expression of miR-29 family members	Qi, (2018)
	*Astragalus* oral liquid	Clinical experiment	Reduce the levels of hs-CRP, TNF-α, and IL-6	Lu et al. (2019)
	*Astragalus* injection	Clinical experiment	Reduce inflammatory factors(TNF-α and IL-6)	Zhang and Zhi, (2014)
	Xinkang Granule	*In vivo*	Decrease the miRNA-21 signalling pathway activation	Liu et al. (2018b)
		*In vivo*	Regulate miRNA-1 and miRNA-133/caspase-3 expressions	Liu et al. (2019)
	Qili Qiangxin Capsule	*In vivo*	Upregulation of miR-133a expression	Chen, (2019)

## Summary: Traditional Chinese Medicine is a Promising Treatment Strategy for Myocardial Fibrosis

Increasing evidence suggests that traditional Chinese medicine is an alternative source of anti-fibrosis drugs and a promising research hotspot. The present review concludes that the role of traditional Chinese medicine in treating cardiac fibrosis and the related experimental performance is reflected mainly in the following perspectives: 1) Traditional Chinese medicine treatment can inhibit the RAAS to prevent cardiovascular diseases; 2) Traditional Chinese medicine can not only improve the histopathology of heart tissue but also restore effective myocardial perfusion and improve heart function; 3) Traditional Chinese medicine can effectively inhibit the proliferation and differentiation of CFs and regulate the growth cycle of fibroblasts, thereby reducing myocardial collagen deposition; 4) Traditional Chinese medicine can regulate various pro-fibrosis factors, cytokines, and cardiovascular active substances, including TGF-β1, CTGF, Ang II, TNF-α, interleukin, NOX2, IFN-r, IL-β, and hs-CRP, and further regulate the activity of related signal pathways to improve MF. 5) Traditional Chinese medicine plays a role in inhibiting myocardial inflammatory reaction, oxygen free radicals, and lipid peroxidation; 6) Traditional Chinese medicine can participate in the metabolic process of ECM by regulating the balance of MMPs/TIMPs and exert the anti-myocardial fibrosis effect. 7) Traditional Chinese medicine can regulate the TGF-β1/Smad signalling pathway by downregulating TGF-β 1 expression, reducing collagen deposition in the heart, and inhibiting MF and ventricular remodelling. 8) Traditional Chinese medicine can regulate myocardial miRNA and inhibit fibroblast proliferation and collagen synthesis.Owing to its multi-component, multi-target, and multi-level characteristics, traditional Chinese medicine can be applied to treat different fibrosis and cardiovascular diseases in different stages. Additionally, traditional Chinese medicine can improve the fibrosis of other organs, providing a new strategy to develop anti-fibrosis drugs.

Owing to its multi-component, multi-target, and multi-level characteristics, traditional Chinese medicine can be applied to treat different fibrosis and cardiovascular diseases in different stages. Additionally, traditional Chinese medicine can improve the fibrosis of other organs, providing a new strategy to develop anti-fibrosis drugs.

Although remarkable progress has been made in exploring the in-depth mechanism of Chinese medicine in preventing and treating MF, the research on anti-myocardial fibrosis effect of Chinese medicine is in its infancy and limited due to the following problems and shortcomings: 1) Research on the anti-myocardial fibrosis mechanism of Chinese medicine has not been extensive and is limited to the RAAS, cardiovascular active substances, inflammation and oxidative stress, and collagen degradation system. However, a few studies have attempted to explore the mechanisms on the molecular level and based on intracellular signal transduction pathways. 2) Many types of MF animal models exist, and the experimental model for exploration of MF pathogenesis mechanism needs a precise and reasonable scientific research design. 3) Although various treatment methods are available, such as invigorating qi, nourishing yin, promoting blood circulation, removing blood stasis, inducing diuresis and detumescence, softening and resolving hard masses, benefiting temperature and yang, promoting blood circulation, and dredging collaterals, no comparative study of each method has been conducted yet. Moreover, a pharmacological comparative study of commonly selected drugs in each method is lacking. 4) Clinically, patients’ symptoms and manifestations are different, and there are different TCM syndromes. However, a unified TCM syndrome differentiation system is also lacking. In addition, the clinical research sample is small, and the repeatability of many therapies and prescriptions is poor. There are only a few prospective, multicentre, and large-sample-controlled studies. 5) Some traditional Chinese medicine formulae, extracts, and monomers of traditional Chinese medicine have unclear specific medicinal components and effective sites, and their target points are unknown.

With the further development of modern molecular biology, the research on the treatment of MF with traditional Chinese medicine has made rapid progress, which shows that traditional Chinese medicine exhibits various advantages and characteristics in treating pulmonary fibrosis. This review shows that TCM and its active ingredients, compound preparation can effectively reduce inflammatory reaction, alleviate oxidative stress, inhibit cardiac fibroblast activation, reduce extracellular matrix, regulate the renin–angiotensin–aldosterone system, downregulate TGF-β1 expression, regulate the TGF-β1/Smad signalling pathway, and regulate microRNA, thereby protecting the heart from injury. Research on the prevention and treatment of MF with traditional Chinese medicine is mostly based on experiments, which provides some objective basis for clinical drug selection. However, the specific effective parts of Chinese medicine and its molecular mechanisms are not clear enough, and there is relatively little research on the corresponding theoretical basis of Chinese medicine. In conclusion, MF is still a thorny clinical challenge. Traditional Chinese medicine has good therapeutic potential for fibrotic diseases. However, it is necessary to further explore the methods of prevention and treatment of MF with Chinese herbs such as *Angelica sinensis* to benefit patients with clinical cardiovascular diseases.
